# Phosphorylation: The Molecular Switch of Double-Strand Break Repair

**DOI:** 10.1155/2011/373816

**Published:** 2011-05-18

**Authors:** K. C. Summers, F. Shen, E. A. Sierra Potchanant, E. A. Phipps, R. J. Hickey, L. H. Malkas

**Affiliations:** Department of Pharmacology and Toxicology, Indiana University School of Medicine, Indianapolis, IN 46202, USA

## Abstract

Repair of double-stranded breaks (DSBs) is vital to maintaining genomic stability. In mammalian cells, DSBs are resolved in one of the following complex repair pathways: nonhomologous end-joining (NHEJ), homologous recombination (HR), or the inclusive DNA damage response (DDR). These repair pathways rely on factors that utilize reversible phosphorylation of proteins as molecular switches to regulate DNA repair. Many of these molecular switches overlap and play key roles in multiple pathways. For example, the NHEJ pathway and the DDR both utilize DNA-PK phosphorylation, whereas the HR pathway mediates repair with phosphorylation of RPA2, BRCA1, and BRCA2. Also, the DDR pathway utilizes the kinases ATM and ATR, as well as the phosphorylation of H2AX and MDC1. Together, these molecular switches regulate repair of DSBs by aiding in DSB recognition, pathway initiation, recruitment of repair factors, and the maintenance of repair mechanisms.

## 1. Introduction

The eukaryotic genome is under constant mutational stress through exposure to exogenous and endogenous agents that damage DNA. These external and internal factors damage DNA introducing a wide variety of genetic alterations including: deletions, translocations, and chromosome loss, which can result in cell death. One type of genetic alteration, DNA double-stranded breaks (DSBs), poses a serious threat to cell viability and genome stability if left unrepaired or repaired incorrectly. In order to repair these DSBs, cells have evolved the complex repair pathways: nonhomologous end-joining (NHEJ) and homologous recombination (HR) [1, 2]. In mammalian cells, the majority of DSBs produced by ionizing radiation are repaired by NHEJ [3], whereas DSBs produced by replication fork collapses are repaired by HR [2–4]. The NHEJ pathway occurs mainly in the G0 and G1 phase of the cell cycle by joining DSB ends directly or after limited processing, whereas HR uses a sister homolog as a template for repair during late S and G2 phases [5]. Encompassing these repair pathways, mammalian cells also possess a DNA damage response (DDR). The DDR is responsible for sensing DNA damage and activating signaling cascades necessary to initiate repair and to stall damaged cells until the damage is resolved [6–8]. Regulation of repair proteins in NHEJ, HR, and DDR pathways is key to maintaining proper repair mechanisms and eliminating defects in these pathways that can cause genomic instability and promote tumorigenesis. 

Regulating repair proteins through posttranslational modification, such as phosphorylation, provides molecular switches for regulating DSB repair. Phosphorylation is the addition of a phosphate (PO_4_) group to a protein in order to induce a conformational change that initiates protein activation or deactivation. In DNA repair proteins, phosphorylation has been found to occur on serine and threonine residues, but it can also occur on tyrosine, histidine, arginine, or lysine residues as well. Phosphorylation of DNA repair proteins generally results in activation of the proteins to facilitate DNA repair. The mechanism of reversible phosphorylation in proteins is an important regulatory mechanism for DNA repair pathways.

## 2. NonHomologous End-Joining

**Figure 1:**
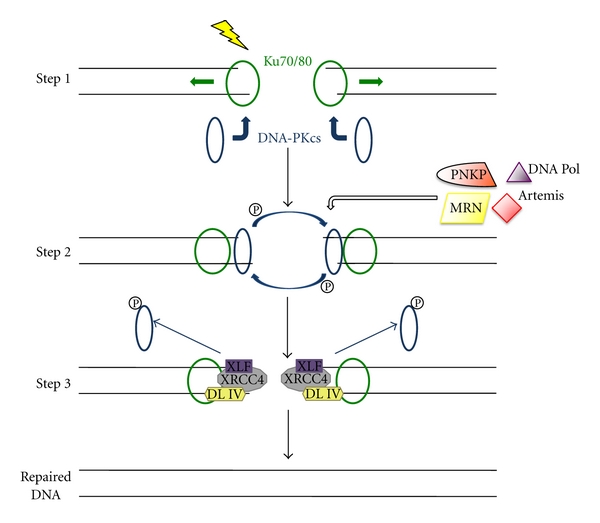
NHEJ model. After a DSB has occurred due to an exogenous or endogenous DNA damaging agent, the NHEJ repair process commences. In step 1, the Ku70/80 heterodimer recognizes and binds the DSB, which induces inward translocation of Ku and recruits DNA-PKcs to the ends of the DSB to form DNA-PK. In step 2, depending on the type and complexity of the DSB break, the DNA ends are processed by different processing factors such as, Artemis, PNKP, DNA polymerases, or the MRN complex (MRE11/RAD50/NBSI). Also, DNA-PKcs autophosphorylate in this second step and will then dissociate from the DSBs. In the final step, XLF stimulates the XRCC4/DNA ligase IV complex to ligate the DNA ends to repair the DSB.

The primary goal of nonhomologous end-joining (NHEJ) is to resolve DSBs generated by exogenous and endogenous agents that damage DNA. Key factors for facilitating proper repair through NHEJ include the Ku70/80 heterodimer, the DNA-dependent protein kinase catalytic subunit (DNA-PKcs), Artemis, X-ray repair complementing defective repair in Chinese hamster cells 4 (XRCC4), DNA ligase IV, and XRCC4-like factor (XLF). As shown in Figure 1, NHEJ proceeds through three key steps: recognition of the break, DNA processing to remove nonligatable ends or other forms of damage at the termini, and finally ligation of the DNA ends [9]. Structural studies of the Ku heterodimer reveal that it exists as a ring structure that binds DNA, interacts tightly with DNA-PKcs forming the protein complex DNA-dependent-protein kinase, DNA-PK [10]. First, the Ku70/80 heterodimer recognizes and binds the DSB, recruiting DNA-PKcs to the ends of the DSB [11] and inducing inward translocation of Ku [12]. DNA-PKcs protects the DNA ends from exonuclease activities and stimulates juxtaposition of DNA ends [13, 14]. Secondly, depending on the type and complexity of the DSB break, the DNA ends are processed by different processing factors such as, Artemis, polynucleotide kinase 3′-phosphatase (PNKP), DNA polymerases, or the MRN complex (MRE11/RAD50/Nijmegen breakage syndrome 1 (NBS1)) [15]. Once bound to the broken ends, DNA-PKcs become activated via autophosphorylation, dissociate from the DSBs, and phosphorylate other targets including replication protein A (RPA), WRN, Artemis, and sometimes H2AX [16–18]. Lastly, XLF stimulates the XRCC4/DNA ligase IV complex to seal the DNA ends [2, 9, 19].

### 2.1. DNA-PK

Researchers have recently implicated DNA-PK as a central regulator of DNA end access via phosphorylation [13]. DNA-PK is composed of DNA-PKcs and the Ku 70/80 subunits, which act as the catalytic and regulatory subunits, respectively [10]. Initially, the Ku subunits recognize DSBs and recruit DNA-PKcs [11], forming the DNA-PK complexes on both ends of the DSB. Assembly of the DNA-PK complex induces a conformational change in DNA-PKcs such that two globular domains interact creating channels that enclose the two DNA ends and protect them from degradation. DNA-PKcs are activated upon association with Ku 70/80 and DNA termini [20]. Next, the Ku heterodimer, which exists as a ring structure [10], translocates to a more interior position on the DNA strand [12]. Finally, autophosphorylation of DNA-PKcs must occur before repair of the DSB [20, 21]. 

The catalytic subunit of DNA-PK, DNA-PKcs, is responsible for the kinase activity. DNA-PKcs is a member of the phosphatidylinositol-3-kinase-like (PIKK) family of serine/threonine protein kinases, which also includes Ataxia Telangiectasia-Mutated (ATM) and ATM- and Rad3-related (ATR) [22]. In response to DNA damage, DNA-PKcs phosphorylation occurs on T2609 which causes colocalization with Ku 70/80 at the DSB site [23]. In order to release DNA termini, DNA-PKcs undergo autophosphorylation on clusters of serine and threonine residues to mechanistically regulate DNA end access during NHEJ. These DNA-PKcs residues are phosphorylated by DNA-PKcs itself and occasionally by ATM or ATR, which causes the release of DNA-PKcs from the DNA and allows NHEJ to proceed [24, 25]. Regulation of DNA-PKcs autophosphorylation and subsequent removal from DNA termini appears to be essential to the continuation of NHEJ. 

Different DNA-PKcs phosphorylation sites have distinct functional consequences. Several studies have discovered two functionally relevant autophosphorylation clusters: ABCDE (T2609, S2612, T2620, S2624, T2638, and T2647) and PQR (S2023, S2029, S2041, S2051, S2053, and S2056) [13, 18]. These sites have been studied by ablating the phosphorylation sites via alanine substitutions or by creating mutants that mimic the phosphorylated sites via aspartic acid substitutions. Phosphorylated mutants with any one or two of the conserved sites mutated to aspartic acid on the ABCDE or PQR clusters are sufficient for NHEJ to proceed. However, mutations that ablate the phosphorylation of all sites of the PQR or ABCDE clusters impart a severe to modest radiosensitive phenotype, respectively. Both cluster mutants are fully functional kinases and are similar to wild-type DNA-PKcs in every aspect except end-processing. Autophosphorylation of the ABCDE cluster promotes end-processing, whereas PQR cluster autophosphorylation inhibits end-processing. Therefore, autophosphorylation of the ABCDE cluster is responsible for DNA-PKcs dissociation from Ku and DSBs, allowing downstream repair factors to access the damaged DNA. Conversely, PQR cluster autophosphorylation retains DNA-PKcs at damaged DNA ends and limits processing of the ends by downstream repair factors. Thus, it appears that autophosphorylation within one cluster opposes the effects of autophosphorylation within the other cluster [13, 21, 26–28]. 

Additionally, DNA-PKcs contains a phosphorylation site within the activation loop (T site) of the kinase (T3950). Phosphorylation of the T site influences the DNA ends joining efficiency [29]. Mimicking phosphorylation at the T site inactivates the kinase and promotes severe defects in both NHEJ and homologous recombination. This suggests that dephosphorylation of DNA-PKcs at T3950 may be required for DNA-PK activity and function. Inhibition of phosphorylation via alanine substitution did not affect DNA-PK protein activity [29]. Mutants with alanine substitutions at the ABCDE cluster, PQR cluster, and T site autophosphorylate at 40% of wild-type levels and undergo autophosphorylation-induced dissociation. This suggests that additional phosphorylation sites are required to regulate DNA-PK function. Recent research has indicated three more phosphorylation sites which appear to play a role in DNA-PK function (S3821, S4026, and T4102); however no clear biological function in NHEJ has been determined [30]. In conclusion, DNA-PKcs undergoes distinct phosphorylation events that determine functional consequences in NHEJ [13, 29, 31].

Several studies have associated full kinase activation with the combined DNA-PK complexes rather than a single DNA-PK complex, suggesting that the DNA-PKcs must phosphorylate only in *trans*. These findings suggest that paired ends of DNA are required before phosphorylation of DNA-PKcs can occur and, subsequently, before other repair enzymes can gain access to damaged DNA ends. This mechanism allows for damaged DNA ends to be protected by DNA-PKcs until the two damaged DNA ends are juxtaposed, and DNA-PK complexes can coordinate with one another to repair the DSBs [13, 14]. Additionally, DNA-PKcs can phosphorylate many other essential substrates *in vitro*, such as RPA, H2AX, Ku70/80 [32], XRCC4 [33], XLF [34], Artemis [30], DNA ligase IV [35], WRN [36], and hnRNP-U [37]. However, phosphorylation of many of these substrates does not appear to be functionally important for NHEJ to repair DSBs *in vivo *[34, 35, 38]. Therefore, the most relevant substrate for DNA-PKcs in NHEJ is DNA-PKcs itself. 

## 3. Homologous Recombination

**Figure 2:**
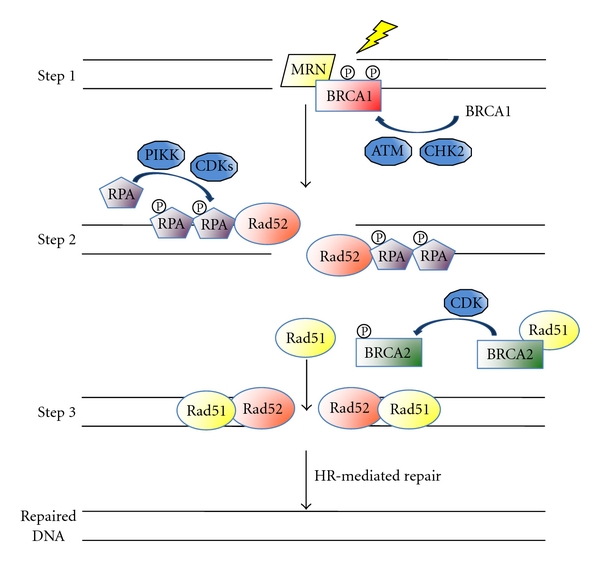
Phosphorylation events in the initiation of HR. After DNA damage is inflicted, the MRN complex processes the ends of the DSBs. BRCA1 is phosphorylated by ATM and CHK2 and regulates the MRN complex. RPA then associates with the 3′ ssDNA overhangs and becomes phosphorylated. Rad52 binds RPA and displaces it to allow for Rad51 binding. BRCA2 binds to Rad51 until BRCA2 becomes phosphorylated, releasing Rad51 and allowing it to localize to the DSB with Rad52. HR-mediated repair continues: Rad51 then forms a nucleoprotein filament that invades a homologous sequence and activates strand exchange to generate a crossover between the juxtaposed DNA.

HR repairs DSBs by using homologous sequences elsewhere in the genome to prime repair synthesis, especially DSBs produced by replication fork collapse [4]. HR is considered 100% accurate for repairing DSBs if the repair template is perfectly homologous. Homologous sequences in the genome can include sister chromatids, homologous chromosomes, or repeated regions on the same or different chromosomes. However, regulation of HR is key to maintaining genomic integrity. As shown in Figure 2, initiation of HR begins with extensive 5′ to 3′ end-processing by the MRN complex, Exo1, and other exonucleases [39]. BRCA1 regulates MRN complex end-processing after phosphorylation and subsequent activation by ATM and CHK2, a checkpoint kinase. Next, the 3′ single-stranded DNA (ssDNA) ends are bound by RPA, which is phosphorylated and actively displaced by Rad52 to allow for Rad51 binding. BRCA2-bound Rad51 is phosphorylated by cyclin-dependent kinases (cdks), releasing Rad51 to bind the ssDNA end, forming a nucleoprotein filament that searches for and invades a homologous sequence. The invading and complementary strands base-pair and extend. Once all intermediates are resolved, any ssDNA gaps and nicks are closed, and the DSB is repaired. HR-mediated repair utilizes the phosphorylation of RPA, breast cancer type 1 susceptibility protein type 1 and 2 (BRCA1 and BRCA2) as molecular switches for regulating DNA repair.

### 3.1. RPA2

Mammalian replication protein A (RPA) is a DNA-binding protein that plays an essential role at replication centers as well as stalled replication forks in the HR repair system. Depletion of RPA leads to persistent unrepaired DSBs [40–42]. At replication centers, RPA acts to recruit and activate key checkpoint mediators [43]. After DNA damage, RPA is recruited to DNA-damage-dependent nuclear foci and interacts with repair and recombination factors to resolve the DSB [44]. RPA is a heterotrimeric protein consisting of RPA1, RPA2, and RPA3 subunits. PIKKs and cyclin-cdk2 complexes regulate RPA via phosphorylation on the extreme N-terminus of the RPA2 subunit [45]. Two of the RPA phosphorylation sites, S23 and S29, are phosphorylated by cyclin-cdk kinases [45], and at least five of the other seven sites (S4, S8, S11, S12, S13, T21, and S33) are phosphorylated in response to ionizing radiation, most likely by PIKKs [46, 47]. To better understand the role of RPA as a molecular switch, RPA2 phosphorylation mutants were created to mimic constitutive phosphorylation. RPA2 mutants that mimic the hyperphosphorylated form are unable to associate with replication centers, but still localize to DNA damage foci. Therefore, following DNA damage, RPA is hyperphosphorylated, limiting DNA replication but aiding DNA repair by marking sites of DNA damage for recruitment of repair factors [40].

### 3.2. BRCA1/2

The breast cancer susceptibility genes (BRCA1 and BRCA2) and Rad51 colocalize to sites of DNA damage and have a role in both the detection and repair of DSBs [48]. BRCA1 binds DNA directly to activate the repair of DSBs and initiate HR. More specifically, BRCA1 regulates the length of ssDNA generation by inhibiting Mre11 activity in the MRN complex. After DNA damage is inflicted, BRCA1 is phosphorylated on the following residues: S1387 by ATM, S988 by CHK2, as well as S1423 and S1524 by other kinases [7, 49, 50]. BRCA2 directly interacts with Rad51 through the BRCT domain on the C-terminus and regulates Rad51 function and localization [51–53]. The carboxy-terminal region of BRCA2 is phosphorylated at S3291 by cyclin-dependent kinases, which blocks C-terminal interactions between BRCA2 and RAD51. Rad51 is then activated to play a major role in HR repair of DSBs [54] by coating ssDNA to form a nucleoprotein filament that invades and pairs with a homologous region in duplex DNA [55]. BRCA1, BRCA2, and Rad51 are important molecular switches for DNA repair that initiate HR.

## 4. DNA Damage Response

In addition to the NHEJ and HR pathways, cells possess the DNA damage response (DDR) which functions as a cellular defense against the accumulation of genetic mutations associated with cancer progression. The DDR safeguards the genomic integrity of cells [56]. After DNA damage is inflicted, cells undergo the DDR leading to the activation of cell cycle checkpoints and apoptosis [6]. Initially, cells deploy groups of DNA damage-sensor proteins to DSBs, which delay cell-cycle progression and activate the correct DNA repair pathways. These DDR proteins relay signals via signal transducers to a set of downstream effectors, which affect cellular events such as repair, cell cycle checkpoint, telomere stability maintenance, transcription control, and apoptosis. Many proteins in the NHEJ pathway, HR pathway, and DDR overlap and have critical roles in repair pathways as well as other biological pathways. 

**Figure 3:**
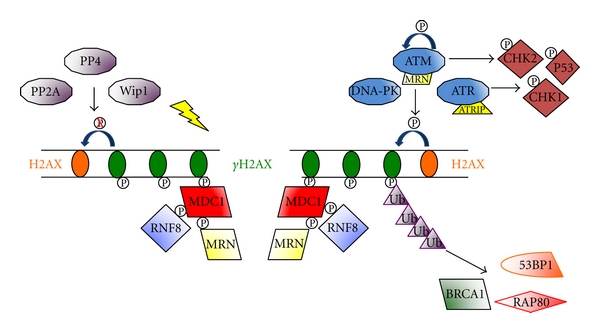
Phosphorylation events in DDR. Key factors in the DDR regulate via a series of phosphorylation events. Members of the PIKK family (ATM, ATR, and DNA-PK) are first to the DNA damage site and phosphorylate histone H2AX. As previously mentioned, DNA-PK is also a critical kinase in the NHEJ pathway. Phosphorylated H2AX recruits and phosphorylates Mediator of Damage Checkpoint protein 1 (MDC1). Once phosphorylated MDC1 then serves as a platform for further recruitment of DDR factors, including the MRN complex and RNF8. RNF8 then ubiquitinates histones and downstream recruits BRCA1, 53BP1, and RAP80.

Regulating the phosphorylation of proteins necessary for DNA repair is key to maintaining proper repair mechanisms [57]. Key factors in the DDR regulate repair via a series of phosphorylation events as seen in Figure 3. Members of the PIKK family (ATM, ATR, and DNA-PK) are first to the DNA damage site and phosphorylate histone H2AX. As previously mentioned, DNA-PK is also a critical kinase in the NHEJ pathway. Phosphorylated H2AX recruits and aids in the phosphorylation of Mediator of Damage Checkpoint protein 1 (MDC1). Once phosphorylated MDC1 then serves as a platform for further recruitment of DDR factors, including NBS1 and RNF8 [57].

### 4.1. ATM and ATR

The PIKKs, ATM and ATR, play a crucial role in DDR by relaying and amplifying the DSB damage signal. In response to DSBs, both ATM and ATR phosphorylate a multitude of substrates, including p53, and the checkpoint kinases, CHEK1 and CHEK2. These phosphorylated substrates promote cell cycle arrest and initiate DNA repair [58]. Arresting the cell cycle allows for enzymes to repair the DNA before DNA synthesis or chromosome segregation initiates. Recruitment of PIKKs to DSBs is required for PIKK-dependent signaling and subsequent DNA repair. ATM and ATR are activated and recruited to the site of DSBs via distinct interacting partners in a phosphodependent manner. 

The activation of the protein kinase ATM initiates phosphorylation of DSB repair and cell cycle control proteins. Initial activation of the ATM kinase remains elusive. However, efficient activation requires the MRN complex and the autophosphorylation of specific ATM serine and threonine residues. ATM is recruited by the MRN complex via an interaction between ATM and the C-terminus of NBS1, a component of the MRN complex [59]. ATM is constitutively present as an inactive dimer and dissociates into monomers once activated by autophosphorylation, acetylation (K3016), and recruitment to sites of DSBs [60–63]. Several studies have revealed five functionally relevant autophosphorylation sites (S367, S1893, S1981, T1885, and S2996) [60–62]. These sites have been studied by creating phosphorylation mutants and by generating phosphospecific antibodies. Phosphorylation mutants at S367, S1893, and S1981 are each defective at ATM signaling *in vivo *and fail to correct radiation-induced cell death [60]. Additionally, phosphorylation mutants at these sites were defective in correcting the S phase checkpoint defect, and phosphospecific antibodies against S367 and S2996 revealed phosphorylation of both sites was rapidly induced by radiation [62]. Therefore, phosphorylation of ATM at these five sites is required for activation of ATM and subsequent phosphorylation of DDR proteins involved in DNA repair and cell cycle control.

Another PIKK, the ATR kinase, phosphorylates ATR substrates in order to inhibit DNA replication and promote DNA repair. Activation of ATR in the DDR requires the recruitment of ATR to RPA-coated ssDNA breaks via an interacting partner, ATR-interacting protein (ATRIP). Disruption of the ATR-ATRIP complex prevents recruitment to the sites of the DSB and leads to defects in repair [43]. Also, ATR activation requires co-localization of the ATR-ATRIP complex with the Rad9-Rad1-HUS1 complex, which creates a heterotrimeric ring-shaped structure that recognizes a DNA end that is adjacent to a stretch of RPA-coated ssDNA. The presence of RPA is required for the binding of the complexes and imparts specificity in loading for the DNA strands giving a preference to the 5′ primer end [59, 64].

In response to DNA damage, the ATM and ATR kinases activate DSB repair enzymes via phosphorylation. ATM and ATR phosphorylates DDR proteins which participate in repair of DSBs and cell cycle control [65]. Most notably, ATM phosphorylates and activates CHEK2 on the SQ/TQ cluster domain containing T68, whereas ATR phosphorylates CHEK1 on S317 and S345 [58, 65, 66]. ATM also phosphorylates many other substrates, including but not limited to MDM2 and MDMX, which influence the stabilization of p53 [65]. Likewise, ATR is capable of phosphorylating BRCA1, Rad17, MCM proteins, RPA, PCNA, and a multitude of other substrates [43, 67]. Although ATM and ATR have preferential phosphorylation targets, both share substrates as well. ATM and ATR both phosphorylate p53, ATM on S15 and ATR on S15 and S37 [68]. Both PIKKs also phosphorylate H2AX, leading to chromatin modification and promoting global genomic nucleotide excision repair [67, 69–71]. 

### 4.2. H2AX

A crucial regulator of the DDR is histone variant H2AX, a member of the H2A family of histones, which packages and organizes DNA into chromatin. H2AX is at the center of cellular responses to DNA DSBs. In response to DNA damage, H2AX is phosphorylated on a conserved serine residue at the carboxyl terminus by PIKK family, including ATM, ATR, and DNA-PKcs. Phosphorylation of H2AX recruits DDR proteins to regions of damaged DNA, leading to delays in the cell cycle and/or DNA repair [72, 73]. The phosphorylated H2AX, *γ*-H2AX, is required for DNA damage signal amplification and the accumulation of DDR factors in subnuclear foci called ionizing radiation-inducing foci (IRIF) [74]. The signal amplification perpetuated by H2AX phosphorylation is not limited to the immediate substrate, but instead spreads to a large chromatin region surrounding the DSB. This major amplification begins with a few damaged base pairs and produces nearly 30 Mbp span modification of chromatin [74]. In addition, DNA DSB repair is enhanced after the phosphorylation of H2AX nucleates the formation of a large complex of factors including MDC1, RNF8, BRCA1, RAP80, 53BP1 [6]. In order to negatively regulate this amplification process, *γ*-H2AX is dephosphorylated by multiple protein phosphatases including PP2A, PP4, PP6, and Wip1. This impressive amplification and regulation of this DDR regulator indicate that H2AX phosphorylation plays a major role in the DDR and the subsequent initiation of DSB repair processes.

### 4.3. MDC1

Another DDR factor, Mediator of Damage Checkpoint protein 1 (MDC1), is a large adaptor protein required for IRIF formation and binds *γ*-H2AX through the BRCT domains. More specifically, MDC1 recognizes a phosphorylation site on the C-terminus of *γ*-H2AX. MDC1 itself is then phosphorylated on the N-terminus. Six repeating residue clusters (SDTDXD/E) within MDC1 are phosphorylated by casein kinase 2 (CK2) to generate recognition sites for Nijmegen breakage syndrome 1 (NBS1) and the rest of the MRN complex (MRE11 and RAD50) [1, 75]. Additionally, E3 ubiquitin ligase, RNF8, is recruited by phosphorylated MDC1 and recruits 53BP1, BRCA1, and RAP80 to damage sites through ubiquitination of *γ*-H2AX. Overall, MDC1 is a key upstream determinant of the DDR that relies on the phosphorylation of H2AX for recruitment as well as phosphorylation of itself to recruit other DDR factors to initiate DNA repair and delay cell cycle progression [1].

## 5. Conclusion and Closing Comments

Together, the reversible phosphorylation of DNA repair factors provides a foundation for a more complete understanding of the role of molecular switches in DNA repair. Regulation of repair proteins through posttranslational modification, such as phosphorylation, provides cells with a mechanism for managing DNA repair processes. NHEJ, HR, and DDR operate together to repair damaged DNA and utilize phosphodependent binding to begin repair, recruit factors to the damage sites, and initiate signaling cascades.
